# Hospital and Institutional News

**Published:** 1916-03-18

**Authors:** 


### HOSPITAL AND INSTITUTIONAL NEWS.

### THE QUEEN VISITS THE FLORENCE NIGHTINGALE HOSPITAL.

THE Florence Nightingale Hospital was honoured last week by an informal visit from Her Majesty the Queen. Her Majesty visited the patients on both the first and second floors, shaking hands with each and saying a few gracious words of sympathy to the patient as well as to any visitor who happened to be present. The Queen also inspected the nurses' sitting and dining rooms, the theatre, bedrooms, kitchen, chapel, and the matron's private rooms; seeing the work proceeding in its normal every-day course in every department. All the staff feel that to have their work recognised and inspected and approved by Her Majesty inspires them to go on with fresh zeal and courage, especially in these difficult times.

### LORD KNUTSFORD.

THE latest report is that Lord Knutsford is making satisfactory progress. The condition arising from the concussion is improving every day. A consultation of the specialists is being held as we go to press to decide when Lord Knutsford may be moved.

### A SEASONABLE BIRTHDAY GIFT.

SIR WILLIAM P. HARTLEY, head of the well-known firm of jam manufacturers, of Liverpool and Bermondsey, has celebrated his seventieth birthday in a manner that might well commend itself to public philanthropists. Sir William announced at a meeting of his London employees on Saturday that he was giving £15,000, invested in 4½-per-cent. War Loan, to twenty-two hospitals in London and seven hospitals in Liverpool. The London, Guy's, Middlesex, and King's College Hospitals will receive £1,000 each; Royal Free, £750; St. George's Hospital, "Waterloo" Hospital, Charing Cross, Westminster, London Temperance, and St. Thomas's Hospitals, £500 each; Evelina Hospital for Children, Metropolitan, London Homœopathic, General Lying-in, Royal Eye, Cancer, and Brompton Consumption Hospitals, £250 each; Queen's Hospital for Children, Hackney, and the Throat and Ear Hospital, Golden Square, £150 each; and the Bermondsey Medical Mission and the Surrey Dispensary, £100 each. A sum of £5,000 will be distributed in Liverpool, the Royal Southern Hospital, the David Lewis Northern Hospital, and the Royal Infirmary receiving £1,000 each, and the Children's Infirmary, Royal Hospital for Women, Stanley Hospital, and Bootle Hospital, £500 each.

### ANOTHER BROWNLOW HILL INFIRMARY SCANDAL.

IN the report on this infirmary, published in THE HOSPITAL of January 1, 1910, page 395, we made a strong appeal to the Guardians to reorganise and change its maternity department, where we found "girls of sixteen and young women physically healthy crowded together in the same wards as older women who may be suffering from contagious diseases of the most repulsive kind." We pointed out the shameful anomaly that the classification of the inmates was a part of the Brownlow Hill system in regard to all except inmates of the maternity ward. It appears from a report in the *Liverpool Evening Express* of an adjourned meeting of the Liverpool Select Vestry on March 7 that none of our suggestions have been followed, and that the scandal in the maternity wards has reached such a high point of degradation that Dr. Evans, in his report, dwelt upon the striking increase in the contagious cases amongst girls from fifteen years upwards, a large number being very young women. His report seemed to indicate that it was practically impossible properly to isolate the cases, and urged, apparently in despair of the vestry's action, that the matter should be brought before the police authorities or the medical officer of health for the city. Brownlow Hill has been a blot upon the reputation of Liverpool in increasing density for some years past, and its condition demonstrates the failure of Poor-Law administration under the Select Vestry, though this city has long been ambitious of being regarded as the second city of importance in the Empire. It would appear to be all a question of money, with an absence of public spirit. Money was never so plentiful in Liverpool as it is in the present day, and though public spirit has been aroused frequently in Liverpool it has never proved strong enough yet to reach the Select Vestry and compel its members to do their duty. They should be made to cease from maintaining a condition of affairs so shocking as to make them a disgrace to the whole nation. Better accommodation for the Lock wards was mentioned, but its provision may depend on a grant being obtained from the Government following the Report of the Royal Commission on Contagious Disease.

### A REMARKABLE EPISODE IN HOSPITAL FINANCE

THE financial policy of the Bristol Royal Infirmary has had a special interest ever since the project, which has resulted in its rebuilding, was decided on. To raise the necessary sum of £137,000 for the new buildings a special fund of £50,000 was raised, and, in addition, investments yielding an income of nearly £4,000 were realised. The result was that only £940 a year remained from this source, and to make good the heavy loss of assured income a policy was devised to secure the largest possible sum from annual subscriptions. This was successful so far that the previous income of £11,000 a year (including dividends) had been re-created largely by annual subscriptions, and, in fact, increased to £15,000 per annum. A bold policy had more or less justified itself; but the expenditure was still in excess of income. The position, therefore, was that £20,000 a year was being spent; that the debt on the new buildings amounted to £25,500, while the accumulated deficit on the Royal Infirmary's income account totalled £14,500. The institution was faced, in other words, with a debt of £40,000 and a considerable annual deficit as well, though against this could be set the imposing new infirmary. A marvellous stroke of good fortune has now occurred in the receipt of £25,000, as a first instalment of a legacy from the late Mr. T. F. M. Capern, which will ultimately amount to £45,000. This most opportune of benefactors left about £130,000, the principal part of which he bequeathed in equal shares to the Royal Infirmary and to the General Hospital, Bristol. Negotiations with the family resulted in the two hospitals receiving the large sum of £45,000 each. It was obviously a matter of financial life or death to the Royal Infirmary that it should do so, but the event has converted a policy partaking of a gamble into a project which has been crowned with success.

### THE HOSPITAL SCULPTOR.

It would be strange indeed if a hospital staff, recruited so largely from artists as is that of the 3rd London General Hospital, Wandsworth, did not produce fruits likely, as the phrase is, to immortalise it in the days to come. Of the illustrations in the hospital's *Gazette* we have had cause to speak in reviewing its earlier numbers. The March issue is specially noteworthy for the reproduction of a bronze bust by Sergeant Derwent Wood, the well-known sculptor, who has found time to exercise his talent in addition to practising what we may call the applied art of modelling, specimens of which from his hands are to be found in the splint room of the hospital. The bust, of which a pleasant photograph appears, is one which the hospital may be proud of, and the C.O., who forms its subject, will have in it yet another reason to congratulate himself on the talented staff of his institution.

### A BATCH OF WAR MAGAZINES.

The March number of the *Southern Cross* (the journal of the 1st Southern General Hospital, Birmingham), and of the *Craigleith Hospital Chronicle*, have now reached this office, together with the February number of the *Ghain Tuffieha Record*, Malta, whose first appearance evoked a warm welcome from us a few weeks ago. If anyone doubts the claims of these war hospital magazines to special attention he would do well to read an editorial note in the last-named journal, in which Colonel Maurice, in recommending a rival paper on the island, remarks that "the war newspaper will have a greatly enhanced value in the days of peace soon to come." In alluding to the verses sent into the same journal the remark is made that "war has always stimulated the poetic faculty." We should be inclined to qualify this. War has often inspired verse in the past, particularly the recollection of war in peace-time; but would it be true to say that the present war has in any but the smallest degree stimulated the poetic faculty? A distinguished professor of literature, who has made poetry his chief study, lately declared that the young generation of writers had "found out war before this inferno started." The latest issue of the *Southern Cross* is rich in illustrations, and also contains in "The Rose Garden," by "N. R.," a lyric of genuine quality. As an instance of the value of an appeal for contributions, the *Craigleith Hospital Chronicle* contains "a heroic episode" from the pen of a Belgian corporal who spent some weeks in a Glasgow hospital. Dr. Gertrude MacLaren recounts her experiences in Serbia, and an account of the Roman hospitals by an Italian contributor is interesting reading. The whole makes an extremely varied number.

### A DOCTOR'S CONSCIENTIOUS OBJECTION.

So far as we have followed their cases before the tribunals, the conscientious objectors have not come very well out of their examination; for they seem to have given very little thought to their alleged "case," and the tribunals have been equally superficial in their questions. Among them, however, is a house surgeon, whose answers will be read with more interest by hospital officials. Dr. G. H. Pearson, house surgeon at the lately notorious Victoria Central Hospital, Liscard, applied on religious grounds for total exemption. The case came before the Wallasey Tribunal, when the applicant's father said that his son's application was quite sincere. At the age of twelve he had been converted under Torrey and Alexander; and at school had desired to devote himself to medical missionary work. He was appointed by the Wesleyans to a Chinese station, but not having been ordained he was refused a passport, and had, therefore, taken his present post. In reply to questions, Dr. Pearson said he would bind up a man's wounds on the battlefield, but would not join the R.A.M.C. or the Red Cross Society, as that meant joining the Army. He would attend soldiers in hospital but not on the battlefield as an Army surgeon; as a civilian, that is to say, but not as a soldier. He was, however, prepared to go out and work under the Serbian Relief Fund in Albania. The Chairman, Alderman Edwin Peace, said that the tribunal could do no more than grant exemption from combatant service. Dr. Pearson declared that he would appeal.

### THE "FICTION" OF PUERPERAL FEVER.

In that seemingly distant past "before the war," but actually not so very long ago, the abolition of compulsory notification as applied to the various morbid states included under the vague term "puerperal fever" was strongly advocated in these columns (The Hospital, July 5, 1913, p. 417). Many reasons were brought forward in support of the proposal; and an exposure of the evasions and futilities of the present system was made. We are pleased to see that the idea is making headway; for in the *Lancet* of March 4 Dr. Fothergill publishes a strong plea for such abolition under the trenchant heading, "The Puerperal Fever Fiction and the Notification Farce." His article is brief and terse, but very much to the point. He has one or two new arguments to add to the familiar old ones, and his reputation both as an obstetrician and as a man of affairs will stand appreciably higher as a result of his logically developed and ably sustained thesis. What arguments there may be on the other side of this question it is difficult to say, for no one ever propounds any. But it seems very difficult to stir up the bureaucrats who control these matters, and nothing gets done. Perhaps Dr. Fothergill's advocacy may help matters on a bit.

### THE IMPORTANCE OF TRIVIAL ILLNESS.

THE importance of what may appear to the uninformed as symptoms of a merely trivial illness is illustrated by an inquest recently held at Batley on a child three years old. The evidence showed that on Sunday the child seemed to have a slight running cold; on Wednesday a sore throat was complained of; early on Friday morning a doctor was summoned, but the child was dead before his arrival. Death was due to diphtheria. The neglect of early symptoms is fortunately not often followed by so tragic a result. It is important, however, that parents should realise that the first symptoms of many serious illnesses are often very slight, and that it is in the early stages of any disease that treatment is most efficacious. The matter is of the greater importance in connection with the infectious diseases, because it is to the slight unrecognised case that the spread of infection is mainly due. When diphtheria is epidemic, as it is at present at Batley, it would be a valuable help in combating the spread of the disease if sanitary authorities would issue leaflets pointing out that the only sign of the disease may be a slight sore throat or a little running at the nose, and that it is of vital importance, both to the individual and to the community, that such cases should be seen at once by a medical man. Only a skilled clinical and bacteriological examination will reveal which cases are diphtheritic and which are not.

### THE HOPE OF THE CRIPPLE.

THE Medical Board has been invited to report on a proposed addition to King Edward VII.'s Hospital, Cardiff, in the shape of a department for the treatment, by hydro- and electro-therapeutics and Swedish methods, of disabled soldiers. The idea is to hasten the return of the wounded; to convert otherwise permanently disabled men into something better; and, after the war, to give the same advantages to those who have been crippled in the course of their employment. Colonel Bruce-Vaughan declared that the proposal really extended an existing department, and that perhaps its chief use would be in safeguarding the health and usefulness of child patients. Money is already in hand towards the carrying-out of the project, which has the chief advantage of being as useful when the war is over as it would be when dealing with the wounded at the present crisis.

### THE DISCIPLINE OF PATIENTS IN MILITARY HOSPITALS.

CONSIDERING the numbers of thoughtless people whose idea of honouring the country's wounded heroes is to supply them with large quantities of intoxicants and other deleterious substances, there have been remarkably few public scandals—a testimony partly to the good sense of the soldiers themselves and partly to the discipline maintained in military hospitals. An exception to this satisfactory state of affairs occurred at the 1st Eastern General Hospital at Cambridge recently. A soldier-patient was under sentence of detention for absence from the hospital without leave, and in the morning was found dead: the medical evidence showed that acute alcoholic poisoning was the cause of death. The Coroner who investigated the case tried very hard to unravel the circumstances in which the soldier had contrived to secure the drink, and cross-examined the sergeant of the guard at some length without success. The evidence of the latter was very unsatisfactory, but he would not admit any knowledge of the affair; and the puzzle remained unsolved. At all military hospitals this particular evil crops up, sooner or later, and the only way to avoid it is to preserve a very strict discipline—stricter than may often appear to the outside public as either necessary or desirable.

### A HISTORICAL PAMPHLET.

IT is always pleasant to hear of long-standing members of hospital staffs who devote their leisure to compiling a history or memoir of their institution. Any recruit to this still too much neglected branch of hospital literature is to be welcomed, and we therefore note with pleasure that Dr. Muriel, whose connection with the Whitehaven and West Cumberland Infirmary has been a long one, has compiled a memoir relating to the institution. We welcome the receipt of all hospital histories, large or small, as a means of encouraging, by a review, less enterprising institutions to realise the value a hospital history has in attracting local support and in preserving a tradition.

### AN ISOLATION HOSPITAL WITHOUT A WATER SUPPLY.

IT might be interesting to learn details of the inception of the isolation hospital which serves the Lynton Urban District Council. According to the report of the medical officer of health, Dr. H. J. Edwards, this hospital seems to have been placed in a most unsuitable spot. Although the number of cases admitted yearly is small, the administrative difficulties are of no light order. In the first place there is the astonishing circumstance that there is no water supply available in the locality where the hospital is situated. In spite of repeated representations on the part of the M.O.H., no steps have been taken to remove the hospital to some place where a water supply can be laid on. In addition to this extraordinary defect the site of the building appears to be not easy of access, and is, in fact, remote enough for the telephone company to refuse to connect it with their circuit. The difficulty of communicating with the doctor when serious cases are admitted is so obvious that even the provision of the suggested cottage for a caretaker seems hardly a sufficient remedy. This building for infectious diseases, if not fulfilling all the desiderata of an isolation hospital, is at least a well-" isolated " institution!

### HOSPITAL FOR LIMBLESS WARRIORS, GLASGOW.

DEFINITE progress is being made with the scheme for the establishment in the neighbourhood of Glasgow of a hospital for limbless sailors and soldiers. At the present time men who have lost their limbs in the war can only be treated at Queen Mary's Auxiliary Hospital, Roehampton, where the accommodation is now more than overtaxed, something like 1,600 cases being on the waiting list for admission. It has long been felt that sailors and soldiers from Scotland should not be compelled to travel all the way to Roehampton after amputation to be fitted with an artificial arm or leg, and the Lord Provost of Glasgow and an influential provisional committee have obtained from Surgeon-General Sir Alfred Keogh, the Director-General of Medical Services, an assurance that the proposed hospital will enjoy the same privileges with regard to grants, etc., and receive the same recognition from the naval and military authorities as the Roehampton Hospital enjoys. The West of Scotland Hospital will in no sense be an adjunct of Roehampton, but a separate and distinct institution amenable only to the naval and military authorities.

### SIR ALFRED JONES MEMORIAL HOSPITAL, GARSTON.

THE connection of the late Sir Alfred Jones's name with hospital work, and especially with tropical medicine, lends an interest to the new institution bearing his name which has been opened at Garston. Known as the Sir Alfred Jones Memorial Hospital, a building in the Georgian style, erected at a cost of £10,000, the accommodation of some twenty beds is divided between two large and two smaller wards. Thanks to the legacy left by Sir Alfred Jones, the plan for establishing such an institution has been completed after being under consideration for more than thirty years. Almost immediately after the opening the first patient was received. The chairman of the committee is Captain Turner. The architects of the building are Mr. C. G. Anderson and Mr. R. P. Crawford, of Liverpool. The post of secretary to the hospital is being filled by Dr. Paterson.

### A HOSPITAL SECRETARY AS MAJOR.

THE list of hospital secretaries on active service would not be complete without mention of the secretary of the Whitehaven and West Cumberland Infirmary, Mr. (now Major) Bewlay. In fact, his absence on active service—which led to Mr. Sand's appointment as honorary secretary till he was compelled to resign for business reasons—has led Major Bewlay to resign his post. Meanwhile, pending presumably another appointment, Mr. G. A. B. Hill is understood to be acting as honorary secretary to the institution.

### A ZEPPELIN WARD.

THE capital of manufacturing England, always comprehensive in its schemes and elaborate in its organisation, has devised a detailed plan for dealing with any "problems" which may be created by the casual visit of a Zeppelin to the city. The chief point in this—as interesting to record for the benefit of future generations as the stories of local precautions against "Boney" have become to us—is the reservation of the vacant wards at the neighbouring joint hospital for the treatment of any persons who may receive injuries as a result of a Zeppelin raid. Indeed, what may be described more accurately as a block has been set apart for this purpose. This is the central point of what is probably the most comprehensive scheme of its kind yet adopted in any industrial area.

### THE WEST HAM AND EASTERN GENERAL HOSPITAL.

THE vast strides forward made by this institution in recent years are perhaps not so fully appreciated in London institutional centres as, on the facts, they certainly should be. At the recent annual general meeting Mr. T. A. Cook, who was chairman for fourteen years in succession and resigned two years ago, quoted some figures connected with the work of the institution which reveal a fine record of progress. Various rebuilding operations, upon which £50,000 in all were spent during his tenure of office, naturally resulted in a great increase of *clientèle* owing to improved conditions. The annual income and expenditure rose from about £5,000 in 1901 to about £13,000 in 1914. The sixty beds of 1902 have become at the present time 110 permanent and 30 temporary lent for the accommodation of wounded soldiers; but Mr. Cook has large visions, for he insists that there ought to be 500. A new nurses' home has been a pressing want, which has just been filled by the munificence of Sir Edwin and Mr. E. H. Tait. Mr. Lyle, the president of the institution, was absent from the meeting through illness; but appreciative references were made to his energy in raising funds. Evidently this hospital has generous friends, although it is rather far from the limelight of central London or of the West End.

### SIR F. MILNER AND THE PERTH HOSPITALS.

SIR FREDERICK MILNER, Bart., on his recent visit to the Perth Hospitals first went to the V.A.D. Hospital in York Place, and was met at the institution by Miss Wilkinson, the matron; Miss Bulloch, county director, Perthshire Red Cross; Dr. Menzies, medical officer in charge; and Mr. F. H. Noad, commandant Perth Men's V.A.D. A little souvenir from their Majesties in the shape of a tin of cigarettes was presented to each soldier by Sir Frederick. The Royal Infirmary, where thirty wounded soldiers are at present under treatment, was afterwards visited by Sir Frederick, who was met at the institution by Lord Provost Scott, chairman of the directors; Mr. Alexander Macduff, of Bonhard, vice-chairman; Mr. James McEwen, chairman of the house committee; Mr. Gavin Morton, a director; Mr. R. Martin Bates, secretary to the directors; the matron, the house surgeon, and a number of the nurses. Several of the men were in bed, but the majority paraded in the ward set apart for the wounded. After the Royal message and the presents of cigarettes had been delivered, Quartermaster-Sergeant Clark, of the Royal Engineers, thanked Sir Frederick for the great interest he took in the welfare of the wounded soldiers, and asked him to convey to their Majesties the men's gratitude. Sir Frederick subsequently visited the hospital at the Queen's Barracks, where he was met by Lieut. Meade, R.A.M.C., and Miss Blackett, hon. secretary Perthshire Red Cross Society.

### THE EAST SUSSEX TUBERCULOSIS SCHEME: SUCCESSFUL OPPOSITION.

Following the adjourned discussion by the East Sussex Insurance Committee on the proposed comprehensive scheme for the treatment of tuberculosis by the County Council, another meeting was held on March 7, a month later than the one referred to in The Hospital recently [for March 11]. At this meeting the opposition to the proposal to hand over the control of insured tuberculosis persons to the County Council was strong enough to defeat the resolution submitted by Mr. W. Austin Hubbard "that the report from the tuberculosis sub-committee . . . be received and adopted." Doubts were expressed as to what the insured person had to gain if the machinery of the Insurance Committee were transferred to the Health Committee of the County Council, and it was feared that the insured person would not receive the extra nourishment that had hitherto been granted by the Insurance Committee, nor that personal attention which he received at the present time. In spite of a declaration by Mr. Hubbard that everything would not be handed over to the County Council, and that the machinery of the committee would be exactly the same as at present, only three members voted in favour, as against nine dissentients.

### ABERDEEN OPEN-AIR CHILDREN'S WARD.

Pending the completion of the new Sick Children's Hospital at Aberdeen, an interesting experiment has been carried out in the grounds of the temporary premises at Kepplestone House, now occupied by the hospital. At a total cost of £650 a new open-air building has been erected in these grounds. This ward is a single-storey structure of wood 88 feet by 20 feet and 8 feet high to the eaves. In the centre is a closed and heated room for washing and dressing patients and for nursing service. The two lateral open wards have a front to the sun which can be half-closed only by pulling shutters up from the floor; at the back, which is walled in, are windows readily opened and ventilators which cannot be closed placed high up under the eaves. It is proposed to move this ward to the new hospital if it prove to be successful administratively and clinically. In fact the new buildings themselves may be partly constructed on the lines of open-air wards. Obviously, a considerable saving in expense would result thereby.

### TUBERCULOSIS PATIENTS.

An important practical point in the administration of sanatorium benefit was brought forward at a recent meeting of the Batley District Insurance Committee. This was the serious leakage which occurred among patients entitled to and recommended for sanatorium benefit by their panel doctor; in many cases those who went to the tuberculosis officer and received forms of application for treatment never troubled the sanatorium sub-committee. Hence a certain number of cases were overlooked. Possibly there are still some persons who misconceive the position; but surely now there can be few who confuse the benefits for which they have in part paid with the notion that they are accepting hospitality from a charitable institution. The point, however, of the situation is that there is a defect in the administration in this district. The fact that a certain patient has received a form of application should obviously be noted, and if no claim is made during a reasonable interval, inquiries should automatically be made. Such cases must not be allowed to drift to their own undoing.

### SANATORIUM BENEFIT IN LONDON.

Attention has been drawn in these columns already to the unsatisfactory state of the sanatorium benefit as available for insured persons in various parts of the country. Insurance Committees, it cannot be doubted, would like to do for the tuberculous insured all and more than all Mr. Lloyd George promised a few years ago. They never could do all that, even before the present financial breakdown became plainly apparent; and now they are compelled—to the indignation of some who have not envisaged the entire situation—to fall further short than ever of the rhetorical fancies of six years ago. The London Committee, for instance, found itself £6,300 out of pocket over sanatorium benefit for 1915; luckily a sum was available from the General Purposes Fund which nearly covered the deficit. For 1916, however, they are faced with a prospect of an additional loss of over £15,000, and with no prospect of help from the General Purposes Fund: that is, they are budgeting for a deficit of a good deal more than £20,000 in sanatorium benefit alone. They quote with approval their medical adviser's dictum that "with limited funds much that is theoretically requisite and actually desirable is practically impossible"; and the problems for consideration are held to be "not what is required for the control and prevention of tuberculosis and the most satisfactory treatment of insured persons, but what is the best way of laying out a limited amount of money."

### ECONOMICAL SCHOOL INSPECTION IN CORNWALL.

By taking advantage of the practical experience and skill of trained nurses in dealing with school children, the medical officer of health for Cornwall, Dr. E. M. Clarke, hopes to be able to cope with the essential parts of the work of school inspection in the county in spite of the altered conditions due to the war. Arrangements have been made for a nurse to visit each school and to record obvious defects; she will also pick out those children who should be referred to the medical officer for further advice from those who, in her opinion, or in that of the head-teacher, need no further examination. By such a curtailment of the routine work Dr. Clarke will probably be able to visit all those schools in which there are seriously defective children and to advise parents as to treatment. Early examination of children apparently needing spectacles will be carried out at Truro. Under the circumstances, this system of inspection is likely to prove sufficiently effective. It has the merits of being economical and time-saving.

### INCREASED COST OF MAINTENANCE.

The Wandsworth Board of Guardians have made investigations into the great increase in the cost of maintenance of the patients in their two infirmaries during the last quarter over the amounts in the same quarter in the previous year. At St. James's Infirmary the total cost was £6,176 for 644 patients, whilst last year it was £3,836 for 543 patients. Canon Curtis, the chairman of the Board, stated that this was due to the fact that meat had gone up 55 per cent. in price, and that other commodities had shown an almost similar increase. The cost at St. John's Hill Infirmary was £4,490 for 458 patients, whilst in 1914 it was £4,223 for 605 patients. The weekly cost in provisions alone went up at St. John's Hill Infirmary from 3s. 8d. per patient to 5s. 2d., and at St. James's Infirmary from 4s. to 5s. 3d. The Board expressed its approval of the explanations given by the staff for these large increases, which, in view of the great rise in prices, could not be remedied.

### DEATHS BY STARVATION OR PRIVATION.

The return recently issued by the Local Government Board of the number of deaths in England and Wales in 1914 due to starvation or accelerated by privation, shows a gratifying decrease upon the average of the last few years. The return gives a total of sixty-two such deaths in England and Wales, of which twenty-five occurred in London. The most striking feature in the London figures is the marked improvement that has taken place in Whitechapel. As we pointed out in The Hospital for May 29, 1915, this parish has previously had by far the worst record of all the London parishes in this respect. In the year under review, however, only one death due to starvation was recorded within its boundaries. Both Shoreditch and Bethnal Green had four such deaths, whilst Poplar, as usual, had none. Of the deaths in London no fewer than six occurred amongst old-age pensioners. This points strongly to the necessity for more supervision of the recipients of these pensions.

### THE LUXURY OF PATENT MEDICINES.

In view of the general inadequacy of the sums available for the treatment of tuberculous persons under the Insurance Act, it is more than ever desirable that the funds should be administered as economically as possible, having regard to the welfare of the patients. One luxury that could be dispensed with to the advantage of the funds, and certainly not to the disadvantage of the patient, is the prescribing of proprietary medicines; and it is satisfactory to note that this view is held by at least some of the Insurance Committees. At Preston lately it was reported to the Committee that a practitioner had prescribed a proprietary compound costing five shillings, the equivalent of which could be obtained for ninepence; and, while the Committee agreed that there should be no misapprehension on the point that insured persons were entitled to the best treatment, they decided that in future such items should not be allowed to be charged upon the Sanatorium Benefit Fund. This policy naturally meets the views of the Panel Committee, and it is to be hoped that if there are any Insurance Committees which have not determined upon the same line of action as the Preston Committee they will lose no time in doing so. To prescribe unnecessarily expensive medicines for a private patient is bad, but to debit public funds with proprietary compounds for which there are equally good substitutes at an eighth the price is worse.

### THIS WEEK'S DRUG MARKET.

The continued demand for drugs for the Medical Services of our own and our Allies' Armies keeps the market busy. Again most of the price changes have been in an upward direction, although in some cases prices are not quite so firmly maintained. For instance, the accumulated stock of Persian opium has imparted a somewhat easier tone to this market. The supply of cocaine immediately available is small, and consequently quotations are rather higher; but when the expected further supplies arrive, it is unlikely that the present high figure will be maintained. Chloroform is dearer, and a further advance is not improbable. Fancy prices are still quoted for cod-liver oil, but the volume of business is by no means large. As a result of very heavy buying of hexamine, the lower price tendency has ceased, and rather higher prices are asked. Formaldehyde is dearer, in consequence of an increased demand. Makers of bismuth preparations have raised their quotations as a result of an advance in the price of the metal; but as they are unable to accept orders for prompt delivery, except in special cases, those who hold stocks can obtain prices considerably above those quoted by the makers. Potassium chlorate is difficult to obtain. Acetanilide and phenacetin are slightly dearer, but otherwise there are no noteworthy changes in synthetic drugs. The offer of a small quantity of aspirin of Australian manufacture is interesting as indicating that Australia has turned its attention to the manufacture of synthetic drugs, but the quantity offered is small and cannot affect market prices in the slightest. Strychnine is dearer. The prices of tartaric and citric acids still have an upward tendency. At the recent public sale of drugs in Mincing Lane high prices were paid for senna leaves, colocynth pulp, and gum elemi; balsam Peru was rather cheaper.

